# Targeting mitochondrial RNA polymerase in acute myeloid leukemia

**DOI:** 10.18632/oncotarget.6129

**Published:** 2015-10-15

**Authors:** Fernando N. Bralha, Sanduni U. Liyanage, Rose Hurren, Xiaoming Wang, Meong Hi Son, Thomas A. Fung, Francine B. Chingcuanco, Aveline Y. W. Tung, Ana C. Andreazza, Pamela Psarianos, Aaron D. Schimmer, Leonardo Salmena, Rebecca R. Laposa

**Affiliations:** ^1^ Department of Pharmacology and Toxicology, Faculty of Medicine, University of Toronto, Toronto, Ontario, Canada; ^2^ Princess Margaret Cancer Centre, University Health Network, Toronto, Canada; ^3^ Department of Psychiatry, Faculty of Medicine, University of Toronto, Toronto, Ontario, Canada; ^4^ Centre for Addiction and Mental Health, Toronto, Ontario, Canada

**Keywords:** acute myeloid leukemia, mitochondria, mitochondrial RNA polymerase, electron transport chain, oxidative phosphorylation

## Abstract

Acute myeloid leukemia (AML) cells have high oxidative phosphorylation and mitochondrial mass and low respiratory chain spare reserve capacity. We reasoned that targeting the mitochondrial RNA polymerase (POLRMT), which indirectly controls oxidative phosphorylation, represents a therapeutic strategy for AML. POLRMT-knockdown OCI-AML2 cells exhibited decreased mitochondrial gene expression, decreased levels of assembled complex I, decreased levels of mitochondrially-encoded Cox-II and decreased oxidative phosphorylation. POLRMT-knockdown cells exhibited an increase in complex II of the electron transport chain, a complex comprised entirely of subunits encoded by nuclear genes, and POLRMT-knockdown cells were resistant to a complex II inhibitor theonyltrifluoroacetone. POLRMT-knockdown cells showed a prominent increase in cell death. Treatment of OCI-AML2 cells with 10-50 μM 2-*C*-methyladenosine (2-CM), a chain terminator of mitochondrial transcription, reduced mitochondrial gene expression and oxidative phosphorylation, and increased cell death in a concentration-dependent manner. Treatment of normal human hematopoietic cells with 2-CM at concentrations of up to 100 μMdid not alter clonogenic growth, suggesting a therapeutic window. In an OCI-AML2 xenograft model, treatment with 2-CM (70 mg/kg, i.p., daily) decreased the volume and mass of tumours to half that of vehicle controls. 2-CM did not cause toxicity to major organs. Overall, our results in a preclinical model contribute to the functional validation of the utility of targeting the mitochondrial RNA polymerase as a therapeutic strategy for AML.

## INTRODUCTION

A critical feature of acute myeloid leukemia (AML) is that it exhibits high oxidative phosphorylation, elevated mitochondrial mass, and reduced spare reserve capacity of the mitochondrial respiratory chain [1-4]. Mitochondrial transcription indirectly controls oxidative phosphorylation since the mitochondrial genome encodes crucial subunits of the electron transport chain [5]. Mitochondrial RNA is relatively abundant, accounting for 5% of the total messenger RNA in most cell types [6], and is transcribed from the mitochondrial genome by the mitochondrial RNA polymerase (POLRMT). POLRMT is a single-subunit RNA polymerase structurally related to the RNA polymerase of bacteriophage and structurally unrelated to other mammalian RNA polymerases [7].

POLRMT is overexpressed in most haematologic malignancies [8], and overexpressing the mitochondrial RNA polymerase increased tumour growth in a xenograft model of breast cancer [9], suggesting that POLRMT contributes functionally to tumour growth. A number of nucleoside analogues used as antiviral agents to target viral RNA polymerases demonstrate off-target inhibition of POLRMT [10]. One of these, 2-*C*-methyladenosine (2-CM), was employed in the current study as a small molecule inhibitor of mitochondrial transcription. The inhibition of mitochondrial translation as a therapeutic strategy for leukemia has shown preclinical efficacy [1] and has progressed to Phase I clinical trials (Clinicaltrials.gov identifier NCT01332786). In the current work, we asked whether inhibiting POLRMT either with shRNA or with 2-CM would also have potential anticancer properties in AML cells.

## RESULTS

### POLRMT knockdown decreases POLRMT protein levels

**Figure 1:**
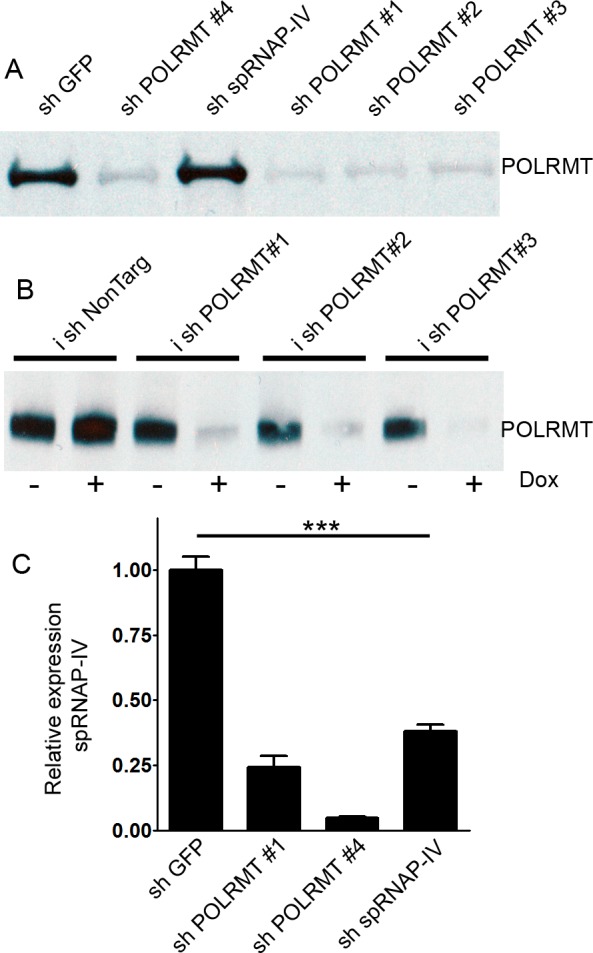
Effects of shRNA against POLRMT on target knockdown **A.** Western blot of POLRMT levels after treatment of OCI-AML2 cells with shGFP, shPOLRMT or sh spRNAP-IV **B.** Western blot of POLRMT levels in OCI-AML2 cells treated with doxycycline-inducible shNonTargeting or shPOLRMT in the absence or presence of doxycycline **C.** Relative expression of spRNAP-IV transcript, normalized to 18S, in OCI-AML2 cells treated with shGFP, shPOLRMT or sh spRNAP-IV.

shRNA delivery of sequences directed against POLRMT was achieved using lentiviral infection in OCI-AML2 cells. Western blotting revealed that the levels of the 135 kD POLRMT protein were reduced in cells infected with four different shRNAs targeting POLRMT (Figure 1A). In order to better control the kinetics of the shRNA-mediated knockdown, the same shRNA sequences were cloned into a single-plasmid doxycycline-inducible system [14]. Doxycycline decreased the levels of POLRMT protein in the doxycycline-inducible system (Figure 1B). The POLRMT gene also has been reported to express an alternative transcript [13] encoding single polypeptide RNA polymerase IV (spRNAP-IV). shRNA against spRNAP-IV reduced the levels of spRNAP-IV transcript (Figure 1C). However, spRNAP-IV protein was not detectable by Western blotting or immunofluorescence (Figure S1), consistent with a recent report indicating that spRNAP-IV protein is not detectable in human cells [15].

### POLRMT knockdown decreases mitochondrial gene expression

**Figure 2:**
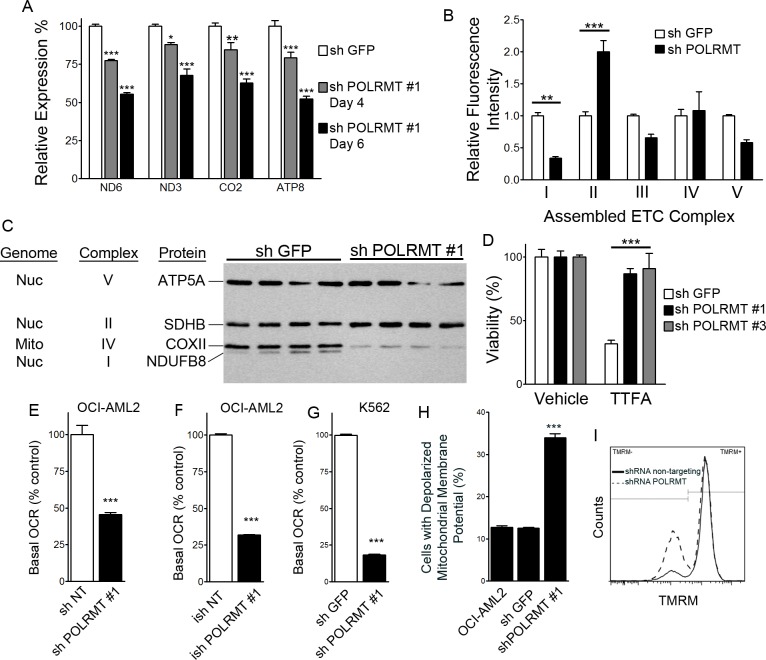
Consequences of POLRMT knockdown on mitochondrial function **A.** Effect of POLRMT knockdown on expression of mitochondrial genes ND6, ND3, CO2 and ATP normalized to 18S, expressed relative to shGFP controls at 4 or 6 days post-infection. **B.** Relative amount of assembled ETC complexes I, II, III, IV and V in OCI-AML2 cells treated with shGFP or shPOLRMT. **C.** Western blotting of individual ETC subunits in lysates from OCI-AML2 cells infected with shGFP or shPOLRMT. **D.** Relative viability of OCI-AML2 cells treated with shGFP or shPOLRMT and the complex II inhibitor TTFA or vehicle. **E.** Basal respiration (oxygen consumption rate, OCR) in OCI-AML2 cells infected with shRNA against a non-targeting control or shPOLRMT. **F.** Basal respiration rate of OCI-AML2 cells treated with inducible shRNA against a non-targeting control or POLRMT, and treated with doxycycline. **G.** Basal respiration rate of K562 cells treated with shRNA against POLRMT or GFP **H.** The proportion of cells with a depolarized inner mitochondrial membrane, in OCI-AML2 cells treated with shGFP or shPOLRMT **I.** Frequency histogram of TMRM- (left peak) and TMRM + (right peak) OCI-AML2 cells treated with shGFP (solid line) or shPOLRMT#1 (dashed bars).

The mitochondrial RNA polymerase transcribes the mitochondrial genome in polycistronic transcripts. Of the 13 protein-encoding mitochondrial genes, we chose to assess the four mitochondrial genes ND6, ND3, CO2 and ATP8 since this set includes a transcript from the light strand promoter (ND6) and from the heavy strand promoter 2, from which the remaining 12 mRNAs are produced. We chose to include mtDNA-encoded subunits of complex I (ND6, ND3), complex IV (CO2) and complex V (ATP8). As a positive control for decreased mitochondrial gene expression, we treated OCI-AML2 cells with ethidium bromide (0.04 μg/mL) for 3 hours, a relatively low concentration that inhibits the expression of mitochondrial genes ND1 and CO2 by at least 50% [16]. Expression of all four mitochondrial genes was significantly reduced by ethidium bromide (Figure S2). In OCI-AML2 cells, POLRMT knockdown had no effect on the expression of 18S ribosomal RNA, and data were normalized to 18S. POLRMT knockdown resulted in the decreased expression of all four mitochondrial genes relative to shGFP controls (Figure 2A).

### Effect of POLRMT knockdown on electron transport chain proteins

We simultaneously measured assembled forms of ETC complexes I-V by employing a magnetic bead multiplex assay that utilizes antibodies raised against assembled ETC complexes [17]. In POLRMT-knockdown cells, the level of assembled complex I was significantly reduced. In contrast, the level of assembled complex II, the only ETC complex comprised entirely of subunits encoded by nuclear DNA, was two-fold higher than in shGFP-infected cells (Figure 2B). Western blotting with antibodies directed against subunits of each complex showed that POLRMT knockdown reduced the levels of a mtDNA-encoded complex IV subunit (mtCOX-II) and a nuclear DNA-encoded complex I subunit (NDUFB8), and increased the level of a nuclear DNA-encoded complex II subunit (succinate dehydrogenase B) (Figure 2C). The functional relevance of the observed elevation in complex II was tested by treating POLRMT-knockdown cells with a complex II inhibitor, theonyltrifluoroacetone (TTFA) (Figure 2D). shGFP-treated cells were sensitive to 100 nM TTFA, while shPOLRMT-treated OCI-AML2 cells were resistant, consistent with the elevated levels of complex II in shPOLRMT-treated cells observed by the multiplex and Western blotting approaches.

### POLRMT knockdown decreases oxidative phosphorylation

In order to determine whether POLRMT knockdown affects oxidative phosphorylation, we measured basal oxygen consumption in intact cells as an index of oxidative phosphorylation and used an extracellular flux analyzer. In OCI-AML2 cells treated with shPOLRMT #1, basal oxygen consumption was reduced relative to the non-targeting control (Figure 2E). In the doxycycline-inducible system, OCI-AML2 cells infected with doxycycline-inducible shPOLRMT #1 had a basal oxygen consumption rate that was lower than the non-targeting control (Figure 2F). To confirm that the decrease in oxygen consumption rate was not unique to OCI-AML2 cells, K562 cells were also treated with shRNA against POLRMT or a non-targeting control, and a decrease in basal oxygen consumption rate with shPOLRMT treatment was observed (Figure 2G), similar to OCI-AML2 cells.

Mitochondrial membrane potential was assessed with the potentiometric dye tetramethylrhodamine, methyl ester (TMRM) and flow cytometry. Frequency histograms revealed a TMRM-negative peak representing cells with depolarized mitochondrial membrane potential, and a TMRM-positive peak, representing cells with a polarized mitochondrial membrane potential (Figure 2I). In OCI-AML2 cells, POLRMT knockdown resulted in the loss of mitochondrial membrane potential relative to shGFP controls (Figure 2H).

### POLRMT knockdown reduces growth and viability of OCI-AML2 cells

**Figure 3:**
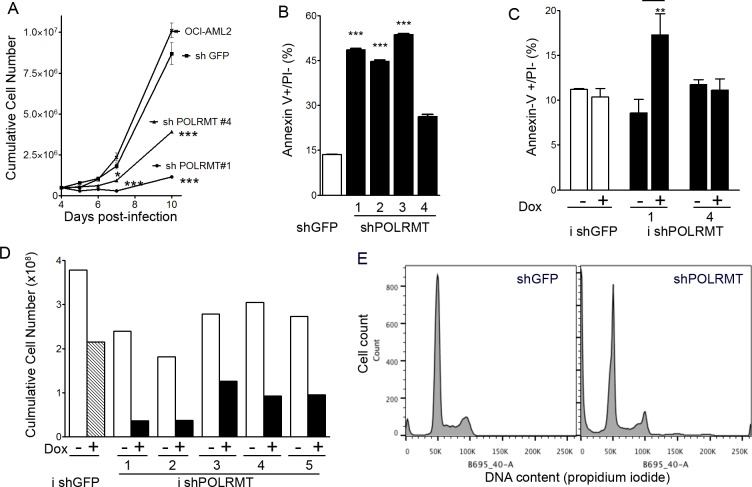
POLRMT knockdown inhibits growth and viability of OCI-AML2 cells **A.** Proliferation curve of OCI-AML2 cells treated with shGFP or shPOLRMT. **B.** The proportion of Annexin V positive/PI negative cells in OCI-AML2 cells treated with shGFP or shPOLRMT **C.** The proportion of Annexin V positive/PI negative cells in cells infected with doxycycline-inducible shRNA against a non-targeting control or POLRMT, and treated with doxycycline or vehicle for 6 days. **D.** Cumulative cell number of OCI-AML2 cells infected with inducible shRNA against a non-targeting control or POLRMT. Cells were treated with doxycycline or vehicle for 10 days **E.** Histograms of propidium iodide staining intensity in OCI-AML2 cells treated with POLRMT shRNA or GFP shRNA for 6 days.

To determine the cellular consequences of inhibiting POLRMT, we assessed the effect of POLRMT knockdown on the growth and viability of OCI-AML2 cells (Figure 3). Proliferation curves revealed that cumulative cell number was lower in POLRMT-knockdown cells relative to shGFP controls (Figure 3A). Early apoptosis, assessed by the proportion of Annexin-V positive/PI negative cells, was measured at 6-7 days post-infection, a time prior to or at which the differences in the proliferation curve were beginning to become apparent. In OCI-AML2 cells infected with four different shRNA constructs against POLRMT, apoptosis was increased up to 3-fold relative to shGFP controls (Figure 3B), indicating that the effects of POLRMT knockdown were more than cytostatic. Proliferation and apoptosis were also measured in the doxycycline-inducible system (Figure 3C, 3D). At 10 days of doxycycline treatment, the cumulative cell number was lower in cells where POLRMT shRNA was induced by doxycycline (Figure 3D). At 6 days of doxycycline treatment, the proportion of early apoptotic Annexin V positive/PI negative cells was elevated two-fold in cells infected with inducible shPOLRMT#1 (Figure 3C). In order to assess whether POLRMT knockdown affected cell cycle progression, nuclear DNA content was assessed by propidium iodide staining and flow cytometry. The proportion of cells in S phase was similar between shGFP controls (11%) and shPOLRMT-treated cells (16%) at 6 days post-infection (Figure 3E).

### POLRMT inhibitor 2-*C*-methyladenosine decreases OXPHOS, growth and viability of OCI-AML2 cells

**Figure 4:**
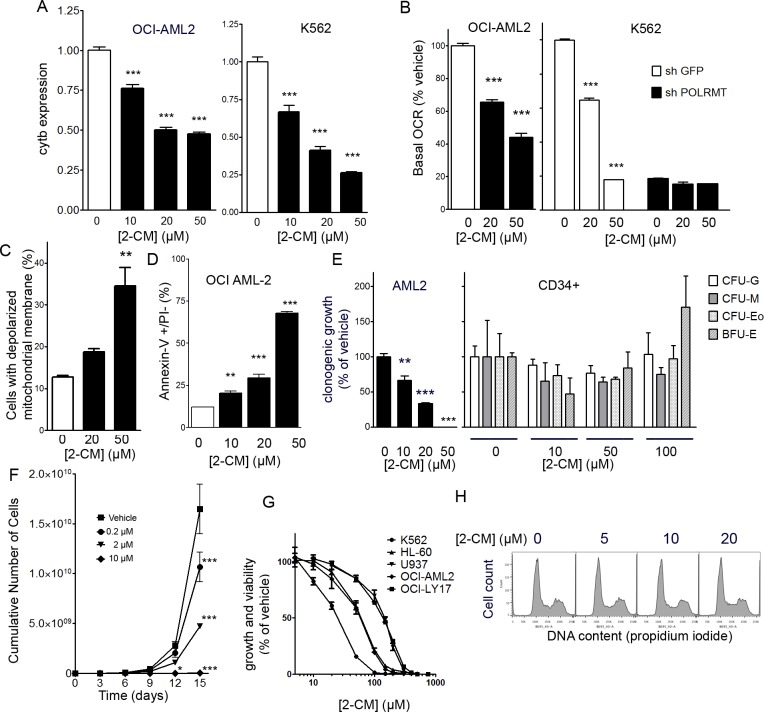
Effects of 2-C-methyladenosine on leukemia cells **A.** Expression of mitochondrial gene cytochrome b normalized to 18S in OCI-AML2 and K562 cells treated with 2-CM for 24 hours. **B.** Basal respiration (oxygen consumption rate) of OCI-AML2 cells treated with 2-CM for 48 hours, normalized to vehicle controls. The basal respiration (oxygen consumption rate) of K562 cells with POLRMT knockdown or shGFP controls treated with 2-CM for 48 hours, values are normalized to shGFP vehicle controls. **C.** The proportion of cells with a depolarized inner mitochondrial membrane, in OCI-AML2 cells treated with 2-CM **D.** Proportion of Annexin V positive/PI negative OCI-AML2 cells treated with 2-CM **E.** Normal human hematopoietic cells (*n* = 3) were treated with 0, 10, 50 or 100 μM 2-CM and plated in clonogenic growth assays. Colonies were counted, including CFU-G, CFU-M, CFU-Eo and BFU-E colony-forming units. Similarly, clonogenic growth of OCI-AML2 cells treated with 0, 10, 20 or 50 μM 2-CM was measured. **F.** Proliferation curve of OCI-AML2 cells treated with 2-C-methyladenosine (2-CM). **G.** Dose-response curves of growth and viability after 2-CM in five leukemia and lymphoma cell lines. **H**. Histograms of propidium iodide staining intensity in OCI-AML2 cells treated with 2-CM for 24 hours.

Several ribonucleoside analogue antiviral drugs designed to inhibit the viral RNA polymerases of the hepatitis C virus or the dengue fever virus exhibit off-target inhibition of the human mitochondrial RNA polymerase, presumably due to similarities in the structures of these RNA polymerases from different species [10]. In OCI-AML2 and K562 cells, 2-CM treatment (10-50 μM) did not affect the expression of 18S ribosomal RNA, and data were normalized to 18S. 2-CM decreased mitochondrial gene expression in a concentration-dependent manner in both OCI-AML2 and K562 cells (Figure 4A). 2-CM decreased basal oxygen consumption in a concentration-dependent manner in both OCI-AML2 and K562 cells (Figure 4B). In K562 cells with POLRMT knockdown, basal oxygen consumption was normalized to shGFP controls; for POLRMT knockdown cells, 2-CM had no effect on the basal oxygen consumption rate, consistent with POLRMT being a relevant target for 2-CM (Figure 4B). In OCI-AML2 cells, 2-CM caused a concentration-dependent loss of mitochondrial membrane potential (Figure 4C) and a concentration-dependent increase in the proportion of early apoptotic cells, as measured by the proportion of Annexin V positive/PI negative cells (Figure 4D). To assess the potential effects of 2-CM on normal human hematopoietic cells, colony forming assays were performed on normal human CD34+ hematopoietic cells. Treatment of human CD34+ cells with 2-CM up to concentrations of 100 μM did not alter clonogenic growth (Figure 4E), while in a parallel clonogenic assay with OCI-AML2 cells, clonogenic growth was impaired at 2-CM concentrations of 10 μM and greater (Figure 4E). To assess proliferation in bulk cultures of OCI-AML2 cells, these cells were treated with 2-CM for two weeks and a concentration-dependent reduction in cell number was observed (Figure 4F). 2-CM concentrations as low as 200 nM reduced the cumulative cell number. In order to assess the efficacy of 2-CM in multiple leukemia and lymphoma cell lines, four additional cell lines were assessed for viability after 2-CM. The IC50 values for 2-CM in K562 (chronic myelogenous leukemia), U937 leukemic monocyte lymphoma, OCI-LY17 (non-Hodgkin lymphoma) and HL-60 (promyelocytic leukemia) were in the mid-micromolar range (Figure 4G). In order to assess the effect of 2-CM on cell cycle, nuclear DNA content was assessed by propidium iodide staining and flow cytometry. The proportion of cells in S phase was similar between vehicle controls (25%) and cells treated with 5 μM (24%), 10 μM (24%) or 20 μM (29%) 2-CM for 24 hours (Figure 4H).

### 2-*C*-methyladenosine decreases tumour growth in OCI-AML2 xenografts

**Figure 5:**
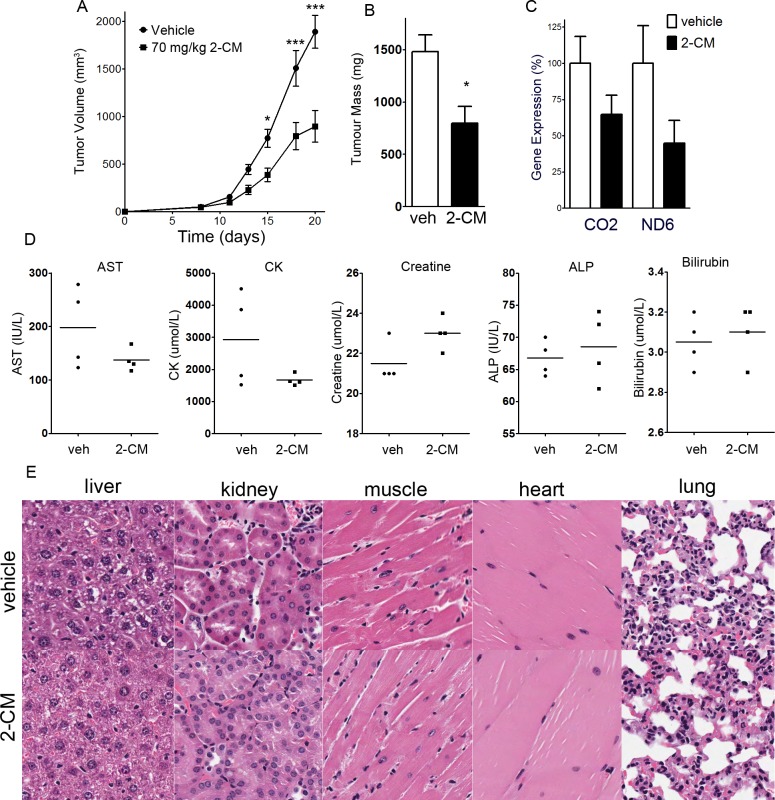
2-C-methyladenosine decreases tumour growth in OCI-AML2 xenografts OCI-AML2 human leukemia cells (5 × 10^5^) were injected subcutaneously into the flanks of male SCID mice. At 11 days after injection, once tumours were palpable, mice were treated with 70 mg/kg 2-*C*-methyladenosine i.p. (*n* = 10) or vehicle (*n* = 10). After 8 days of drug treatment, mice were euthanized, tumours and organs were excised, and tumour mass was measured. **A.** Tumour volume was measured over time. **B.** At the completion of the experiment, tumour mass of excised tumours was measured. **C.** Expression of mitochondrial genes CO2 and ND6 in excised tumours was measured and normalized to 18S. **D.** Plasma levels of asparate transaminase, alkaline phosphatase, creatine, creatine kinase, and bilirubin were measured at the end of the experiment (*n* = 4 per group). **E.** Hematoxylin and eosin staining of sections of liver, kidney, heart, skeletal muscle and lung from tumours of mice treated with 2-CM or vehicle.

Given the efficacy of 2-CM in cell culture, we assessed the efficacy of 2-CM in a xenograft model using published protocols [1]. The maximum tolerated dose of 2-CM was determined to be 70 mg/kg i.p. SCID mice were injected subcutaneously with 5 × 10^5^ OCI-AML2 cells, tumours were allowed to form for 8 days, then mice were injected with 2-CM (70 mg/kg i.p.) or vehicle daily for an additional 11 days, at which time the mice were euthanized. 2-CM decreased tumour volume and tumour mass to approximately half of the vehicle controls (Figure 5A, 5B). RNA was isolated from tumours harvested at the end of the experiment. The expression of 18S was similar in tumours from 2-CM-treated mice and vehicle controls. The expression of the mitochondrial genes CO2 and ND6 were measured and normalized to 18S. The levels of CO2 and ND6 were lower in 2-CM-treated tumours than vehicle controls, but the differences were not statistically significant (Figure 5C). Plasma measurements of liver and kidney function (aspartate transaminase, alkaline phosphatase, creatine, creatine kinase, bilirubin) were unaltered in 2-CM-treated mice relative to vehicle controls (Figure 5D). Histological assessment of major organs revealed no overt toxicity (Figure 5E).

## DISCUSSION

The main conclusions drawn from the results of the current study are that POLRMT knockdown or treatment with 2-*C*-methyladenosine, an inhibitor of mitochondrial transcription, triggers the death of AML cells. Moreover, 2-*C*-methyladenosine showed antileukemic activity *in vivo* in the absence of organ toxicity.

For POLRMT, it has been reported that a minor splice variant, spRNAP-IV results in an N-terminal truncated protein [13, 18]. Recent studies have been unable to replicate the involvement of spRNAP-IV in the transcription of nuclear genes [15]. We were similarly unable to detect spRNAP-IV protein, and shRNA directed against the spRNAP-IV transcript did not affect cell proliferation (data not shown). We conclude that the functionally relevant product of the *POLRMT* gene in OCI-AML2 cells is POLRMT, the mitochondrial RNA polymerase.

POLRMT knockdown or 2-CM treatment decreased mitochondrial gene expression. Since mitochondrial transcription occurs in a continuous polycistronic fashion, transcription of the genes furthest from the transcriptional start site (such as the cytochrome b gene on the mtDNA heavy chain) are predicted to be most affected by the chain terminating action of 2-CM.

In POLRMT knockdown cells, we observed that the mitochondrially-encoded COX-II protein, a subunit of complex IV, was decreased. In POLRMT knockdown cells, the level of assembled complex I was reduced, likely as a result of the high proportion of mtDNA-encoded subunits in complex I [5]. The level of a nuclear-encoded subunit of complex I was also decreased in POLRMT-knockdown cells, as has been observed in cells lacking TFAM, a major transcription factor for POLRMT [19]. We also noted that complex II, an ETC complex composed entirely of nuclear DNA-encoded subunits, was elevated in POLRMT knockdown cells. A similar finding of elevated complex II accompanied by decreases in complex I and sometimes decreases in complexes III-V have been observed in cells in which TFAM has been deleted [19, 20]. The mechanism of complex II elevation is unknown but may relate to an alternative entry point of electrons into ETC via complex II.

We observed that POLRMT knockdown or 2-CM treatment increased cell death without affecting cell cycle. Our results are consistent with a genome-wide RNAi study indicating that in HeLa cells, POLRMT RNAi does not alter cell division but does decrease viability [21].

Several ribonucleoside analogues designed to inhibit viral RNA polymerases such as those of the hepatitis C and Dengue fever viruses inhibit mitochondrial transcription [10]. Some of these ribonucleoside analogues can be triphosphorylated intracellularly. Several of these analogue triphosphates are substrates for POLRMT, and are incorporated by the polymerase into a growing RNA chain, where they then act as chain terminators [10]. In the current study with leukemia cells, the shRNA knockdown of POLRMT or the treatment with 2-CM had similar effects on mitochondrial gene expression, oxidative phosphorylation, apoptosis and cell proliferation, consistent with previous reports that 2-CM inhibits mitochondrial transcription in human cells [10]. We consider 2-CM to be a tool molecule rather a candidate for therapeutic application. One limitation of 2-CM is that it can also inhibits elongation by RNA polymerase II [10]. However, the proofreading ability of TFIIS excises 2-CM from transcripts of RNA polymerase II, and the transcriptional elongation of the RNA polymerase II transcript GAPDH in human cells is unaltered by concentrations of 2-CM up to 200 μM [10]. 2-CM does not affect DNA polymerases alpha, beta or gamma at concentrations up to 50 μM [22], but its potential effects on RNA polymerases I and III are unknown.

POLRMT-knockdown cells and leukemia cells treated with 2-CM showed a decrease in oxidative phosphorylation. This observation is consistent with the reliance of AML cells on oxidative phosphorylation for growth and viability, and the high mitochondrial mass and the low spare reserve capacity of AML cells [23] along with the antileukemic effects of inhibiting mitochondrial translation [1]. Leukemia stem cells are unable to respond to decreased oxidative phosphorylation by increasing glycolysis [4], confirming the functional importance of the reliance on oxidative phosphorylation. Moreover, mutations in mtDNA-encoded subunits of complex IV have been associated with poor disease-free survival in AML patients [24], supporting the importance of mtDNA-encoded electron transport chain subunits in AML.

Results from the colony-forming assays in normal human hematopoietic CD34+ cells suggest the existence of a therapeutic window for 2-*C*-methyladenosine, where OCI-AML2 cells show decreased clonogenic growth at 10 μM, while the clonogenic growth of CD34+ cells was unaffected at concentrations up to 100 μM. In addition, for CD34+ cells, the number of granulocyte-, macrophage- and eosinophil-colony forming units and erythroid burst-forming units was unaltered by concentrations of 2-CM up to 100 μM, suggesting that 2-CM did not affect the differentiation of CD34+ cells along multiple lineages. 2-CM is not toxic to human liver cells at concentrations up to 100 μM [22]. Results from xenograft experiments with OCI-AML2 cells treated with 70 mg/kg 2-CM indicate that 2-CM has antileukemic activity *in vivo*, as measured by reduced tumour volume and tumour mass. These antileukemic effects occurred in the absence of organ toxicity, as measured by serum enzymes and histology of major organs.

In conclusion, these results contribute to the functional validation in cell culture and *in vivo* of the utility of targeting the mitochondrial RNA polymerase as a therapeutic strategy for AML. The strategy of targeting mitochondrial RNA polymerase should theoretically also be more broadly appropriate for other cancer types dependent on oxidative phosphorylation, such as certain types of breast cancer [25, 26], and subset of melanomas and pancreatic cancers [27, 28].

## MATERIALS AND METHODS

### Cell culture

OCI-AML2, HL-60, OCI-LY17, K562 and U937 cells were cultured in IMDM media, 10% fetal bovine serum, and 100 U/mL penicillin and 100 μg/mL streptomycin, all from Life Technologies. For lentiviral production, Lenti-X 293T (Clontech) were grown in DMEM media, 10% fetal bovine serum, and 100 U/mL penicillin and 100 μg/mL streptomycin, all from Life Technologies. For doxycycline-inducible constructs, 293 EMT packaging cells were grown in high-glucose DMEM, 1% Glutamax and 25 mM HEPES (Life Technologies), 10% tetracycline-free FBS (Clontech) as well as 750 μg/ml Geneticin/G418 to maintain EBNA1, 200 μg/ml Hygromycin B to maintain MSR and 250 μg/ml Zeocin to maintain TetR, using antibiotics from Bioshop Canada, Inc. For doxycycline selection, 1 μg/mL (Sigma) was employed. BJ fibroblasts were cultured in DMEM media, 10% fetal bovine serum, and 100 U/mL penicillin and 100 ug/mL streptomycin, all from Life Technologies. The OCI-AML2 and OCI-LY17 cells were kind gifts from Drs. Mark Minden and Hans Messner, respectively (Princess Margaret Cancer Centre, Toronto). The HL60, U937 and K562 cells were kind gifts from Dr. Suzanne Kamel-Reid (Princess Margaret Cancer Centre, Toronto). The HL60, U937 and K562 cells were confirmed by ATCC short tandem repeat (STR) testing (HL60, January 2015; U937 and K562, September 2011).

For proliferation curves, OCI-AML2 cells were infected with shRNA for four days, with puromycin selection starting at 24 hours post-infection. Cells were seeded at 100,000/mL and the number of trypan-excluding cells was counted daily (days 5,6 and 7), reseeded at 100,000/mL and then viable cell number was counted at day 10. For the doxycycline-inducible cells, cells were cultured in 1 μg/mL doxycycline or vehicle and re-seeded every 3 days. At day 10 the cumulative number of trypan-excluding cells was determined.

For colony-forming assays, human CD34+ cells were obtained from Stem Cell Technologies (Vancouver, Canada). CD34+ or OCI-AML2 cells were plated in methylcellulose and appropriate media (Stem Cell Technologies) at clonal densities, treated with 10-100 μM 2-CM and imaged 9 (OCI-AML2) or 14 (CD34+) days later, according to published methods [11]. For CD34+ colonies, colony-forming units-granulocyte (CFU-G), -macrophage (CFU-M), -eosinophil (CFU-Eo) and burst forming units-erythroid (BFU-E) were counted.

### Chemicals

2-*C*-methyladenosine (CAS 15397-12-3) was obtained from Santa Cruz Biotechnology, Inc. (Santa Cruz, California, U.S.A.), MedChem Express (Monmouth Junction, New Jersey, U.S.A.) or Carbosyth Ltd.(Berkshire, UK). Theonyltrifluoroacetone was obtained from Sigma.

### Lentivirus production and infection

Lentivirus expressing shRNA against regions common to both isoforms of POLRMT was generated using the RNAi Consortium clones TRCN0000053114 (targets position 1960 of the coding sequence of POLRMT) termed shPOLRMT#1, TRCN0000333642 (targets position 3309 of the coding sequence of POLRMT), termed shPOLRMT#2 and TRCN0000053113 (targets position 1634 of the coding sequence of POLRMT), termed shPOLRMT#3, that are engineered in the pLKO.1 plasmid (Sigma-Aldrich Oakville, Ontario, Canada). As a negative control, shRNA against green fluorescent protein, in the pLKO.1 vector was employed (Sigma-Aldrich Oakville, Ontario, Canada). In order to generate additional shRNA against POLRMT and spRNAP-IV, the pLKO.1 plasmid [12] was obtained (Addgene plasmid 10878 Addgene, Cambridge, Massachusetts, U.S.A., originally generated by Dr. David Root). The plasmid was cut with AgeI and EcoR1 and the relevant hairpin oligonucleotides were inserted. The oligonucleotides were designed against the published targeting sequences [13] for spRNAP-IV (target region is GGCAAAGAAGGTAACACAA) and for the exon 3 of POLRMT (target region is CAAAGATACTGGAGAAGGATA), termed shPOLRMT#4. Cloning was confirmed by DNA sequencing.

To generate lentivirus, the pLKO.1 plasmids were mixed with plasmids pMD2.G and psPAX2 (Addgene plasmids 12259 and 12260, originally generated by Dr. Didier Trono) and TransIT LT-1 transfection reagent (Mirus Bio LLC, Madison, Wisconsin, U.S.A.) according to the manufacturer's instructions. The plasmid mixture was added to Lenti-X 293T packaging cells (Clontech Laboratories, Inc., Moutain View, California, U.S.A.) and allowed to incubate for 24 hours. Media was then exchanged for media containing 30% fetal bovine serum, and cells were grown for an additional 24 hours. Media was harvested, concentrated 30-fold through 100 kD cutoff centrifugal concentrator columns (EMD Millipore, Billerica Massachusetts, U.S.A.) at 4,000 x g for 20 minutes and frozen at −80 degrees C. For infections, virus was added to OCI-AML2 cells in media containing 5 μg/mL protamine for 24 hours, then media was removed and replaced with media containing 2.5 μg/mL puromycin for 72 hours of selection.

For the doxycycline-inducible shRNA system, a single lentiviral vector [14] plasmid system was employed. Using Gateway cloning, shRNA oligonucleotides were generated by PCR and cloned into the Entry vector pDONR221 using the BP clonase reaction, and transformed into One Shot^®^ OmniMAX ™ 2 T1 Phage-Resistant Cells *E. coli* and selected with 100 ug/mL ampicillin. For the cloned shRNAs, the prefix “i” was added to indicate inducible. Target sequences designed against regions common to both isoforms of POLRMT were (3114, CGGTGGATGTACCCATGCTTT (targets 1984 of coding sequence) i shPOLRMT #1; GACTCCAAGGTCAAGCAAATA (targets 3309 of coding sequence) ish POLRMT #2;

CAACACACGTAAGCAGAAGAA (targets 3386 of coding sequence) ish POLRMT #4; sequence designed against spRNAP-IV: GGCAAAGAAGGTAACACAA (ish spRNAP-IV), or sequence designed against the exon 3 of POLRMT: CAAAGATACTGGAGAAGGATA (targets 424 of coding sequence). A negative control non-targeting shRNA (using Non-Target shRNA #1, Sigma) was also cloned in parallel. Cloning was confirmed by sequencing. Gateway cloning was used (LR clonase reaction) to transfer the shRNA into the desination vector pLV719G; transformants were selected with kanamycin. pLV719G-ShRNA plasmids were mixed with plasmids pMD2.G and psPAX2 (Addgene plasmids 12259 and 12260) and TransIT LT-1 transfection reagent and added to 293EMT. Viral supernatant was concentrated as above. For infections, virus was added to OCI-AML2 cells in media containing 5 μg/mL protamine for 24 hours, then media was removed and replaced with media containing tetracycline-free FBS (Clontech Laboratories, Inc., Moutain View, California, U.S.A.) and 2.5 μg/mL puromycin for 72 hours. To induce the expression of shRNA, infected OCI-AML2 cells were grown in media containing 1 μg/mL doxycycline.

For the overexpression of spRNAP-IV, the vector pLX304 (Addgene plasmid 25890) was employed, and Gateway cloning was used to insert a truncated version of POLRMT (starting with the ATG corresponding to a N262 truncation mutant), using as a template I.M.A.G.E. clone

6572256 that contains the POLRMT cDNA. Lentivirus was produced as above, and HeLa or OCI-AML2 cells were infected and selected with blasticidin.

### Gene expression assays

Total RNA was isolated from infected OCI-AML2 cells using the RNEasy plus kit (Qiagen) or an Aurum kit (Bio-Rad Laboratories, Inc.) Cells were assessed at 4 or 6 days post-infection for the shRNA studies, or at 24 hours after 2-CM. cDNA was synthesized using random priming and Superscript III (Life Technologies.) TaqMan analysis of genes ND3, ND6, CO2, ATP8 and 18S was performed using primers and probes (Applied Biosystems, Inc.) and Taqman Gene expression master Mix (Applied Biosystems) and an Applied Biosystems 7500 Real Time PCR instrument. Standard curves with variable amounts of cDNA input were conducted to confirm linearity of response. Experiments were performed in triplicate and relative expression was determined using the delta delta Ct method. Mitochondrial gene expression was normalized to 18S expression, and data were expressed relative to shGFP controls. RNA content was determined using A260/280 on a Nanodrop spectrophotometer (Thermo Scientific).

For spRNAP-IV expression, primers p2 (Intron 1 forward) GTGGTTTCTTATGCAGCCTC gtggtttcttatgcagcctc and p3 (exon 3 reverse) ATCCTTCTCCAGTATCTTTGC were employed. KiCqStart Master Mix (Sigma) was utilized, with amplification in a BioRad CFX96 Real-time PCR machine, and expression was normalized to 18S.

### Measurement of assembled electron transport chain complexes

The level of assembled electron transport chain complexes was determined using a Human Oxidative Phosphorylation Magnetic Bead Panel Milliplex MAP Kit, according to the manufacturer's (EMD Millipore) instructions. 10 μg of protein lysate from OCI-AML2 cells infected with shGFP or shPOLRMT#1 for 6 days was run in triplicate on a Luminex 200 instrument (EMD Millipore). The levels of nicotinamide nucleotide transhydrogenase were also measured to confirm equal loading of samples.

### Western blotting

2 × 10^6^ OCI-AML2 cells were pelleted and frozen at −80 degrees. For the detection of POLRMT, cells were lysed in RIPA lysis buffer and proteins were separated on a 7.5% PAGE minigel (Bio-Rad Laboratories). For mitochondrial proteins, cells were lysed in 1.5% n-beta-maltoside (Sigma) dissolved in PBS. Lysates were supplemented with Halt protease inhibitors (Thermo Scientific) and 0.5 mM EDTA. For mitochondrial proteins, separation was performed on a 12% PAGE gel. Semi-dry transfer was performed (Trans-Blot SD, Bio-Rad) onto PVDF membranes. Primary antibodies and concentrations were: POLRMT, Abcam ab32988, 1:250; OXPHOS cocktail, Abcam ab110411, 1:1,000 (contains antibodies against complex I, NDUFB8, complex II, SDHB, complex III, UQCRC2, complex IV, COXII, complex V, ATP5A), and alpha-tubulin, 1:1000.

### Flow cytometry

OCI-AML2 cells were treated with shRNA for 7 days, or 2-CM for 3 days. For TMRM studies, OCI-AML2 cells were incubated with IMDM containing 400 nM Tetramethylrhodamine, methyl ester (TMRM) (Invitrogen, Burlington, ON, Canada) for 20 minutes. Cells were pelleted, resuspended in PBS, and analyzed on a flow cytometer. For Annexin staining, OCI-AML2 cells were pelleted, washed in PBS, pelleted, and resuspended in 150 μL binding buffer containing 5 μL of Annexin-V-FITC and 50 μg/mL propidium iodide (BD Pharmingen). Flow cytometry analysis was performed using a FACSCalibur or a BD FACSCanto flow cytometer (BD Biosciences, Mississauga, ON, Canada) and FLOJO software (Tree Star, Ashland, OR, USA). 10,000 events were recorded per sample, and single cells were gated using forward and side scatter. Experiments were performed in triplicates, and repeated twice to ensure reproducibility. For DNA content, cells were fixed with 70% ethanol, stained with propidium iodide, and analysis performed as above. Cell cycle phases were estimated using the Dean-Jett-Fox model and FloJo software.

### Measurement of respiration by extracellular flux analysis

Cellular energy metabolism in intact cells was determined by the measurement of metabolic profiles over time. OCI-AML2 cells were treated with shRNA for 6 days or with 2-CM for 2 days at which time they were resuspended in unbuffered medium, and seeded at 1.2×10^5^ viable cells/well in XF96 plates (Seahorse Biosciences), then incubated for for 45 min at 37°C in a CO_2_-free incubator. Oxidative phosphorylation was measured by the oxygen consumption rate (pMol/min) using the Seahorse XF24 Extracellular Flux analyzer (Seahorse Biosciences, Billerica, MA). Experiments were performed in triplicates, and repeated at least twice to ensure reproducibility.

### Viability assays

5,000 OCI-AML2 cells were seeded into 96-well white plates and cultured with thenoyltrifluoroacetone (TTFA, 100 nM) or DMSO vehicle in IMDM media supplemented with 10% fetal bovine serum and penicillin/streptomycin. 72 hours later, viable cell number was determined using the Cell Titer Glo assay (Promega), a luminescence-based measure of ATP content. Luminescence was read on a luminescence detection plate reader (GloMax, Promega). For dose-response curves, cells were seeded at 5,000 cells/well and cultured with 2-CM for 72 hours.

### Xenograft model

Twenty male SCID mice, 10-12 weeks of age, were implanted subcutaneously with 3 × 10^5^ OCI AML2 cells. Eight days later, mice were randomized into two groups: 10 mice were injected with 70 mg/kg 2-C-methyladenosine, i.p. and 10 mice were injected with vehicle controls (DMSO with cremophor, 10%v/v in 0.9% sodium chloride solution). Mice were injected daily, once a day, for eleven days. Tumour volume was measured with calipers at days 8,11,13, 15, 18 and 19 at which time mice were euthanized. Tumours were weighed, serum was taken, and major organs (liver, kidney, lung, muscle and heart) were harvested. All animal studies were carried out according to the regulations of the Canadian Council on Animal Care and with the approval of the Ontario Cancer Institute Animal Ethics Review Board.

### Statistics

Data were analyzed statistically using one-way ANOVA, two-way ANOVA or t-tests as appropriate and *P* values < 0.05 were considered significant. Data were analyzed using GraphPad Prism (GraphPad Software, Inc.). On graphs, * indicates *P* < 0.05, ** indicates *P* < 0.01 and *** indicates *P* < 0.001.

## SUPPLEMENTARY MATERIAL FIGURES



## Abbreviations

2-CM: 2-C-methyladenosine
AML: acute myeloid leukemia
ATP5A: ATP synthase subunit alpha, mitochondrial
ATP8: ATP synthase protein 8
BFU-E: burst forming units-erythroid
CFU-Eo: colony forming units-eosinophil
CFU-G: colony-forming units-granulocyte
CFU-M: colony forming units-macrophage
CO2: mitochondrially encoded cytochrome c oxidase II
ETC: electron transport chain
mtCOX-II: mitochondrially encoded cytochrome c oxidase II
mtDNA: mitochondrial DNA
ND3: NADH dehydrogenase subunit 3
ND6: NADH dehydrogenase subunit 6
NDUFB8: NADH dehydrogenase 1 beta subcomplex 8
OCI-AML2: Ontario Cancer Institute acute myeloid leukemia 2
OXPHOS: oxidative phosphorylation
POLRMT: mitochondrial RNA polymerase
SCID: severe combined immunodeficiency
SDHB: succinate dehydrogenase complex subunit B
spRNAP-IV: single polypeptide RNA polymerase IV
TFAM: transcription factor A, mitochondrial
TFIIS: transcription factor II S
TMRM: tetramethylrhodamine, methyl ester
TTFA: theonyltrifluoroacetone
UQCRC2: ubiquinol-cytochrome c reducatase core protein II.
