# Supplementary material for: Targeting mitochondrial RNA polymerase in acute myeloid leukemia
Source: Oncotarget. 2015 Oct 15;6(35):37216–28. doi: 10.18632/oncotarget.6129 (PMC4741925; doi:10.18632/oncotarget.6129)
Supplement: Supplementary file 1 [file oncotarget-06-37216-s001.pdf]

Targeting mitochondrial RNA polymerase in acute myeloid leukemia

Supplementary Material

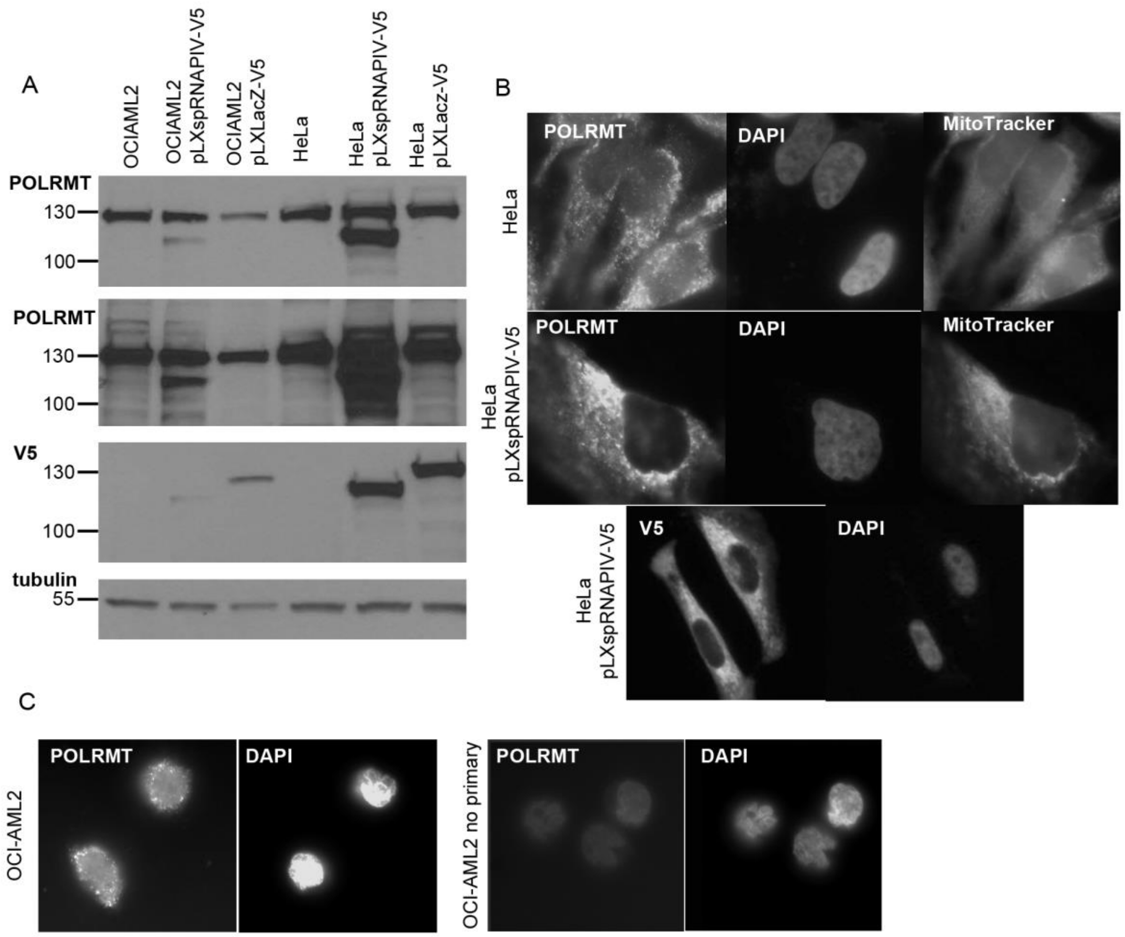

Supplementary Figure 1. Evaluation of POLRMT and spRNAP-IV protein levels and localization. A. Western blotting of whole-cell lysates of OCIAML2 cells, or OCIAML2 cells overexpressing spRNAP-IV-V5 or LacZ-V5, along with whole cell lysates of HeLa cells, or HeLa cells overexpressing spRNAP-IV-V5 or LacZ-V5, probed with an rabbit anti-POLRMT antibody. The predicted molecular weight of the mitochondrial RNA polymerase is 135 kD and the predicted molecular weight of spRNAP-IV is 110 kD. The second image is a longer exposure of the first. The blot was stripped and reprobed with a mouse anti-V5 antibody, followed by stripping and reprobing with the loading control alpha tubulin. **B**. Immunofluorescent images obtained

using POLRMT or V5 antibodies, or MitoTracker or DAPI staining in HeLa cells (top row) or HeLa cells overexpressing spRNAP-IV-V5 (second and third rows) **C**. Immunofluorescent images obtained using POLRMT antibodies in OCIAML2 cells (left) and a technical control where primary antibody was omitted (right)

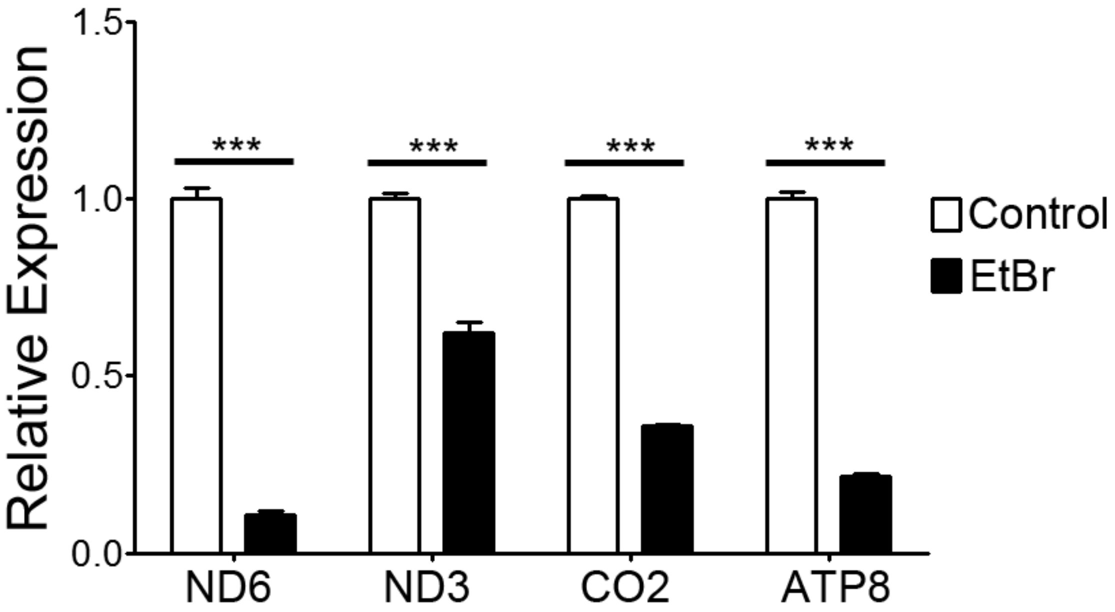

Supplementary Figure 2. Mitochondrial gene expression changes after ethidium bromide.

OCI-AML2 cells were treated with ethidium bromide for 3 hours, and expression of mitochondrial genes ND6, ND3, CO2 and ATP were measured and expressed relative to 18S and plotted relative to vehicle controls.
